# Supplementary material for: The Effect of Amino Acids on Production of SCFA and bCFA by Members of the Porcine Colonic Microbiota
Source: Microorganisms. 2022 Mar 31;10(4):762. doi: 10.3390/microorganisms10040762 (PMC9025589; doi:10.3390/microorganisms10040762)
Supplement: Supplementary file 1 [file microorganisms-10-00762-s001.zip › microorganisms-1589175-supplementary.pdf]

microorganisms

MDPI

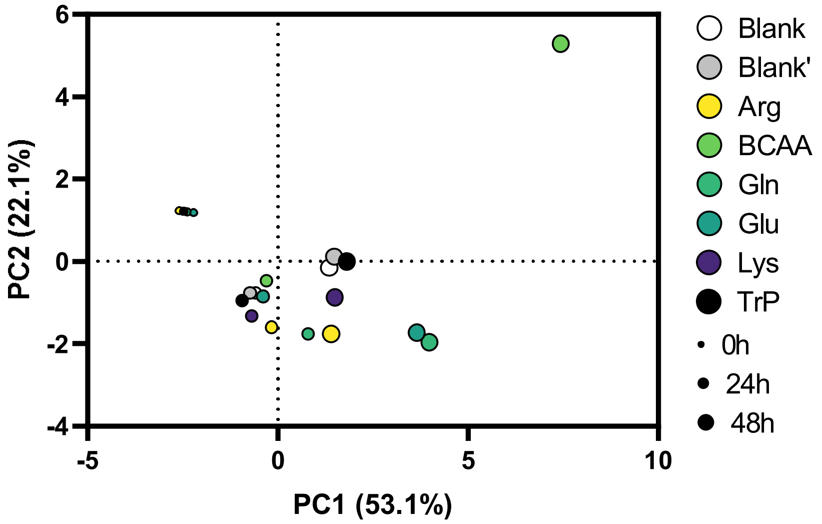

Figure S1. PCA based on microbial metabolic activity on different time points (0h, 24h and 48h) during the incubation of porcine colonic microbiota in presence of various amino acids *versus* the untreated control incubations (Blank and Blank'). The experiment was performed in two different runs. While run 1 consisted of testing blank, Arg, BCAA, Lys and Trp, run 2 consisted of blank', Gln and Glu.

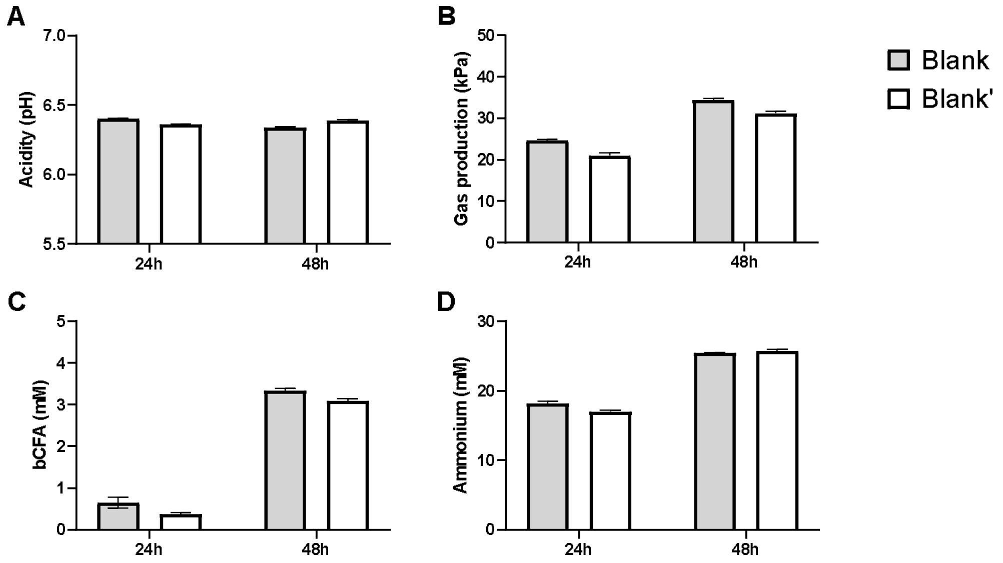

Figure S2. Average (± SD) acidity (pH), gas production (kPa), and markers of proteolytic fermentation (ammonium and bCFA (= sum of isobutyrate, isovalerate and isocaproate)) during both blank incubations (Blank and Blank') with a porcine colonic microbiota (n = 3).

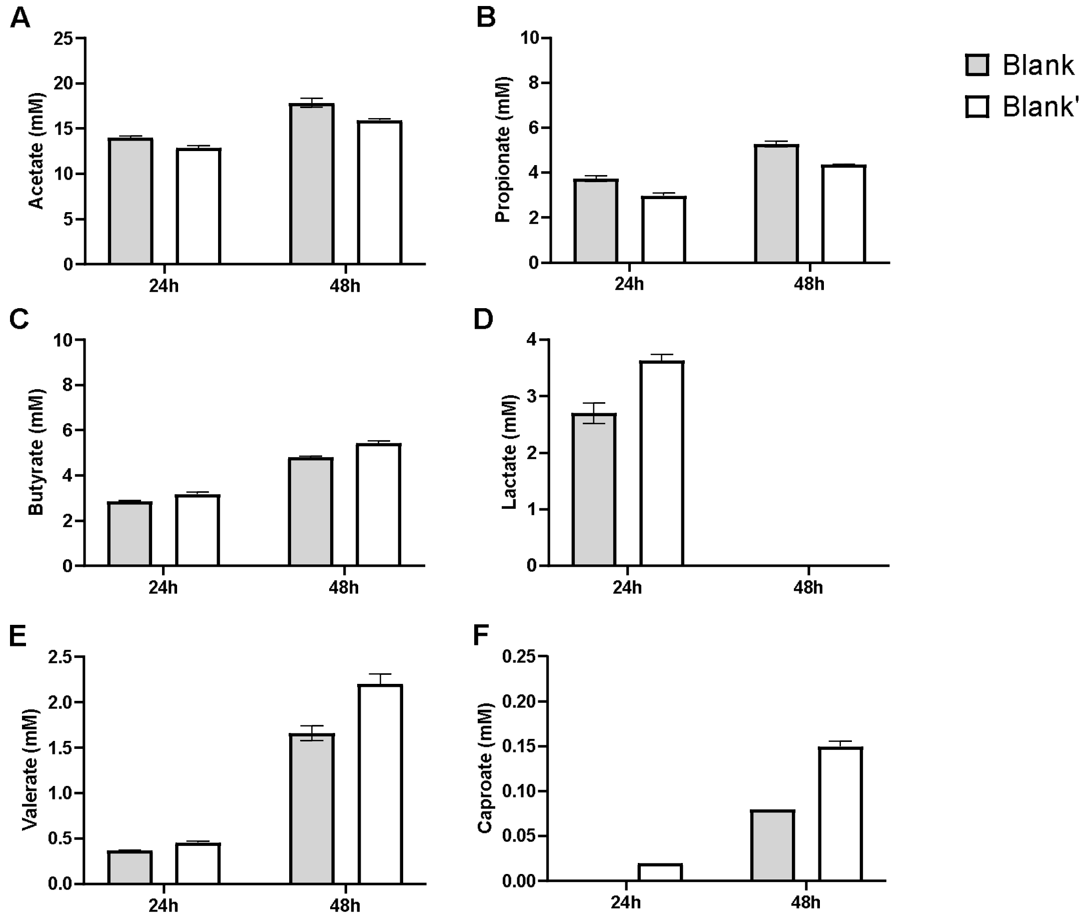

Figure S1. Average (± SD) SCFA (acetate, propionate, butyrate, valerate and caproate) and lactate (mM) levels during both blank incubations (Blank and Blank') with a porcine colonic microbiota (n = 3).

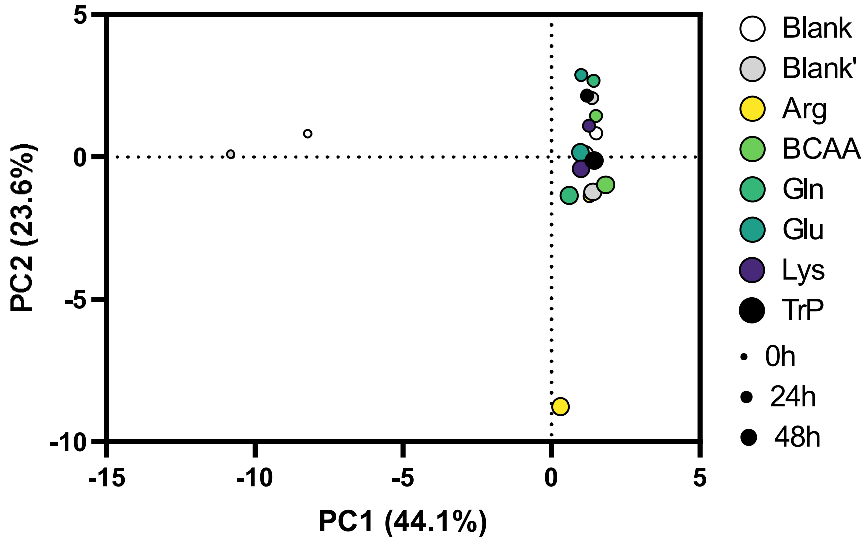

Figure S4. PCA based on microbial community composition (family level; absolute values, estimated upon multiplying with total cell counts (cells/mL)) on different time points (0h, 24h and 48h) during the incubation of porcine colonic microbiota in presence of various amino acids *versus* untreated control incubations (Blank and Blank'). The experiment was performed on two different occasions. While run 1 consisted of testing blank, Arg, BCAA, Lys and Trp, run 2 consisted of testing blank', Gln and Glu.

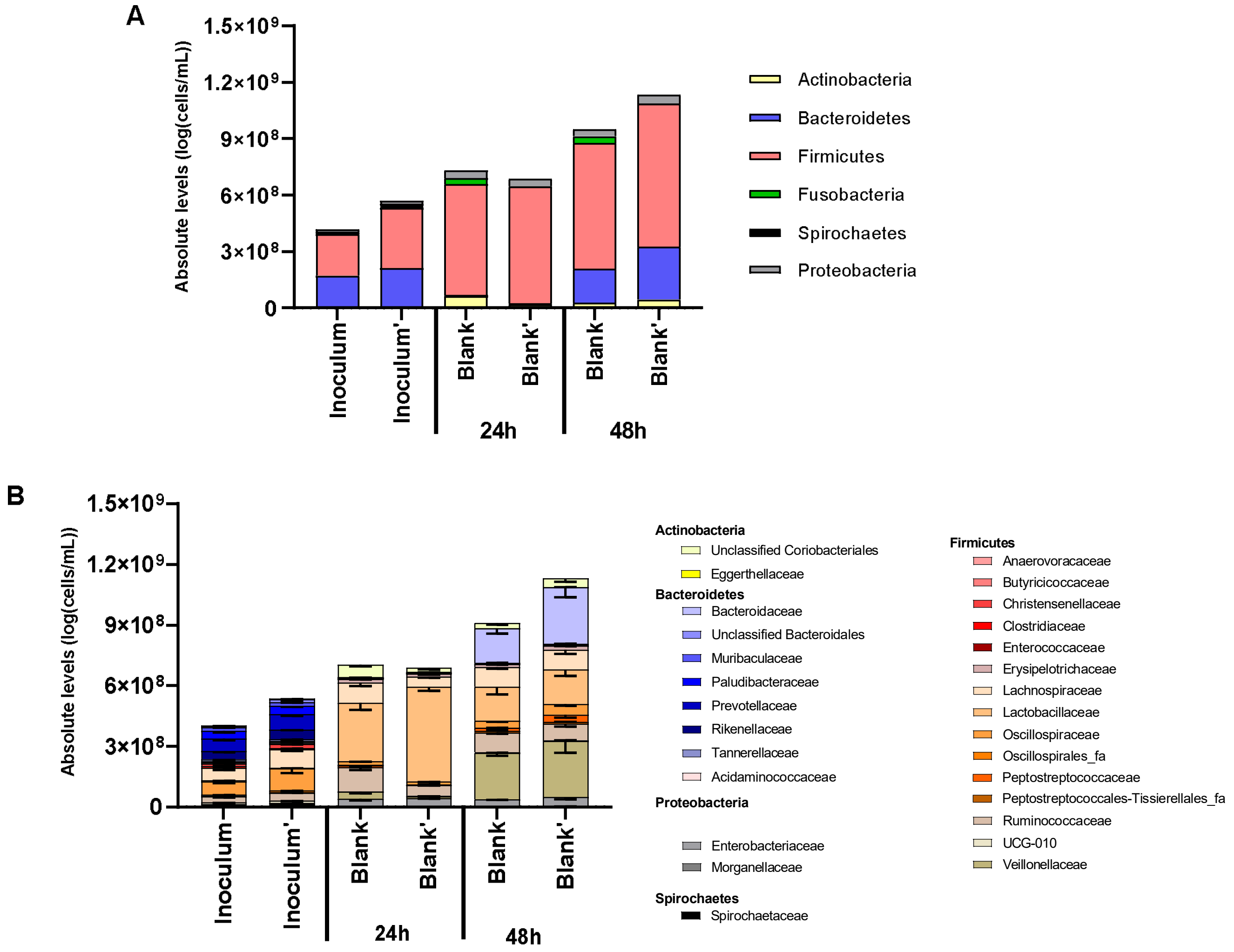

Figure S5. Microbial composition at phylum (A) and family level (B), expressed as absolute values (estimated upon multiplying proportions (%) based on 16S rRNA gene profiling with total cell counts) at the start and after 24h and 48h of incubation during two blank incubations (Blank and Blank')with a porcine colonic microbiota (n = 3). Values are expressed as averages (± SD) measured during the two independent runs performed during the project.

Table S1. Microbial composition at family level, expressed as absolute values (estimated upon multiplying proportions (%) based on 16S rRNA gene profiling with total cell counts) at the start and after 24h and 48h of incubation during two blank incubations (Blank and Blank') with a porcine colonic microbiota (n = 3). Values are expressed as averages (± SD) measured during the two independent runs performed during the project.

Phylum	Family	0h		24h		48h	
		Inoculum	Inoculum'	Blank	Blank'	Blank	Blank'
Actinobacteria	Unclassified *Coriobacteriales*	5.70	5.93	7.79	7.27	7.40	7.63
	Eggerthellaceae	5.60	5.69	5.90	5.69	5.95	6.06
Bacteroidetes	*Bacteroidaceae*	5.55	5.85	6.31	6.58	8.23	8.44
	Unclassified Bacteroidales	6.92	7.15	4.68	4.78	< LOQ	5.11
	Muribaculaceae	7.20	7.31	5.83	5.74	5.36	5.77
	Paludibacteraceae	7.58	7.63	5.10	5.05	5.14	< LOQ
	Prevotellaceae	7.80	7.87	5.70	5.72	5.30	5.75
	Rikenellaceae	7.62	7.67	6.21	6.22	5.90	6.15
	Tannerellaceae	6.86	7.05	5.39	5.35	6.40	5.91
Firmicutes	*Acidaminococcaceae*	6.56	6.64	5.45	5.76	5.77	6.11
	Anaerovoracaceae	6.51	6.71	5.70	5.45	5.81	5.78
	Butyricicoccaceae	6.72	6.71	5.54	5.54	5.67	5.93
	Christensenellaceae	7.09	7.27	5.73	5.83	5.84	6.17
	Clostridiaceae	6.94	6.64	5.87	5.79	6.15	6.03
	Enterococcaceae	< LOQ	< LOQ	4.82	4.93	5.34	5.76
	Erysipelotrichaceae	6.06	6.31	7.26	7.17	7.12	7.25
	Lachnospiraceae	7.80	7.96	8.01	7.69	7.98	8.00
	Lactobacillaceae	6.77	6.50	8.46	8.67	8.22	8.22
	Oscillospiraceae	7.82	8.04	7.27	7.13	7.55	7.72
	Oscillospirales	6.48	6.85	5.31	5.36	5.26	5.47
	Peptostreptococcaceae	6.55	6.40	7.00	6.19	7.17	7.54
	Peptostreptococcales-Tissierellales	< LOQ	< LOQ	5.61	5.67	6.83	7.06
	Ruminococcaceae	7.50	7.60	8.07	7.74	8.00	7.91
	UCG-010	6.90	7.08	5.85	5.72	6.30	6.32
	Veillonellaceae	5.36	5.10	7.55	7.06	8.36	8.45
Proteobacteria	*Enterobacteriaceae*	< LOQ	< LOQ	7.60	7.61	7.57	7.65
	Morganellaceae	< LOQ	< LOQ	5.83	6.12	5.70	6.40
Spirochaetes	*Spirochaetaceae*	7.13	7.29	5.49	5.71	5.45	5.88

Table S2. Effect of amino acids on microbial composition (phylum level; absolute values, estimated upon multiplying proportions (%) based on 16S rRNA gene profiling with total cell counts) after 24h and 48h of incubation with a porcine colonic microbiota (n = 3). The data is presented as the average difference between the log_{10}-transformed absolute abundance (log(cells/mL)) in a treatment *versus* the corresponding untreated blank incubation. A value below zero indicates a decrease upon treatment, while a value above zero, indicates that this phylum is stimulated by a given amino acid. Statistically significant differences as compared to this blank are indicated in bold ($p < 0.05$).

Phylum	24h						48h					
	Arg	BCAA	Gln	Glu	Lys	Trp	Arg	BCAA	Gln	Glu	Lys	Trp
Actinobacteria	-0.03	-0.11	-0.13	-0.08	-0.19	**-0.39**	**0.65**	**0.46**	-0.18	0.07	0.32	**0.30**
Bacteroidetes	**1.23**	-0.23	**-0.39**	-0.33	0.93	**-0.79**	**0.33**	0.00	0.02	-0.09	0.03	-0.04
Firmicutes	0.01	0.00	0.04	-0.11	-0.06	**-0.24**	-0.04	0.06	0.06	0.11	-0.05	-0.12
Proteobacteria	0.01	-0.10	-0.19	**0.23**	0.03	**0.42**	**0.34**	0.05	-0.01	**0.48**	**0.21**	**0.79**
Spirochaetes	0.03	0.02	-0.22	-0.11	-0.07	-0.37	0.34	-0.02	0.13	0.06	-0.05	-0.33

Table S3. Effect of amino acids on microbial composition (family level; absolute values, estimated upon multiplying proportions (%) based on 16S rRNA gene profiling with total cell counts) after 24h of incubation with a porcine colonic microbiota (n = 3). The data is presented as the average difference between the log_{10}-transformed absolute abundance (log(cells/mL)) in a treatment *versus* the corresponding untreated blank incubation. A value below zero indicates a decrease upon treatment, a value above zero, indicates that this family is stimulated by a given amino acid. Statistically significant differences as compared to this blank are indicated in bold ($p < 0.05$).

Phylum	Family	Arg	BCAA	Gln	Glu	Lys	Trp
Actinobacteria	Unclassified *Coriobacteriales*	-0.09	-0.12	-0.11	-0.11	-0.17	**-0.40**
	Eggerthellaceae	**0.94**	-0.01	-0.10	0.10	-0.12	**-0.20**
Bacteroidetes	*Bacteroidaceae*	**1.62**	-0.31	-0.71	**-1.93**	-0.85	**-1.41**
	Unclassified Bacteroidales	0.26	0.03	-0.09	0.29	0.15	-0.21
	Muribaculaceae	**1.37**	-0.16	-0.10	-0.10	**0.66**	**-0.72**
	Paludibacteraceae	-0.35	-0.35	-0.16	0.22	0.21	-0.18
	Prevotellaceae	-0.32	-0.26	**-0.23**	0.06	0.02	**-0.66**
	Rikenellaceae	-0.38	-0.23	**-0.24**	0.02	-0.01	**-0.65**
	Tannerellaceae	**0.87**	-0.26	-0.01	-0.26	-0.06	-0.42
Firmicutes	*Acidaminococcaceae*	**0.57**	-0.45	0.08	0.15	0.14	-0.36
	Anaerovoracaceae	-0.28	-0.29	0.16	-0.17	0.05	-0.12
	Butyricicoccaceae	-0.11	-0.23	0.07	0.32	-0.12	-0.24
	Christensenellaceae	0.10	-0.04	-0.06	0.05	0.05	-0.28
	Clostridiaceae	0.24	-0.20	-0.25	0.08	0.01	-0.22
	Enterococcaceae	1.57	0.35	-0.01	-0.28	-0.03	-0.08
	Erysipelotrichaceae	0.07	-0.25	-0.23	**-1.70**	-0.07	**-0.74**
	Lachnospiraceae	0.09	**-0.22**	**-0.30**	**-0.57**	-0.06	**-0.51**
	Lactobacillaceae	0.00	-0.02	-0.08	-0.16	-0.02	-0.09
	Oscillospiraceae	**0.13**	**-0.23**	**0.34**	0.02	-0.04	**-0.29**
	Oscillospirales	0.09	-0.46	-0.12	-0.26	-0.03	-0.44
	Peptostreptococcaceae	-0.42	-0.22	-0.36	-0.31	-0.60	-0.49
	Peptostreptococcales-Tissierellales	0.32	-0.38	-0.53	**-1.17**	-0.47	-0.61
	Ruminococcaceae	**-0.16**	-0.09	**0.51**	**0.19**	-0.10	**-0.37**
	UCG-010	-0.07	**-0.33**	-0.24	-0.02	0.10	**-0.61**
	Veillonellaceae	0.23	**0.55**	**0.24**	**0.42**	0.20	**-0.98**
Proteobacteria	*Enterobacteriaceae*	-0.01	-0.09	-0.19	**0.24**	0.12	**0.43**
	Morganellaceae	0.05	0.15	-0.60	-1.06	-0.18	-0.11
Spirochaetes	*Spirochaetaceae*	0.01	0.04	-0.22	-0.12	0.04	-0.35
